# Cortical morphology of the pars opercularis and its relationship to motor-inhibitory performance in a longitudinal, developing cohort

**DOI:** 10.1007/s00429-017-1480-5

**Published:** 2017-07-29

**Authors:** Lauren B. Curley, Erik Newman, Wesley K. Thompson, Timothy T. Brown, Donald J. Hagler, Natacha Akshoomoff, Chase Reuter, Anders M. Dale, Terry L. Jernigan

**Affiliations:** 1 Department of Cognitive Science, University of California, San Diego, La Jolla, USA; 2 Center for Human Development, University of California, San Diego, 9500 Gilman Drive, La Jolla, CA 92093-0115 USA; 3 Department of Psychiatry, University of California, San Diego School of Medicine, La Jolla, USA; 4 Center for Multimodal Imaging and Genetics, University of California, San Diego School of Medicine, La Jolla, USA; 5 Department of Neurosciences, University of California, San Diego School of Medicine, La Jolla, USA; 6 Department of Radiology, University of California, San Diego School of Medicine, La Jolla, USA

**Keywords:** Motor inhibition, Cortical surface area, Cortical thickness, Magnetic resonance imaging, Brain development, Inferior frontal gyrus

## Abstract

This study investigates the relationship between variability in cortical surface area and thickness of the pars opercularis of the inferior frontal gyrus and motor-inhibitory performance on a stop-signal task in a longitudinal, typically developing cohort of children and adolescents. Linear mixed-effects models were used to investigate the hypotheses that (1) cortical thinning and (2) a relatively larger cortical surface area of the bilateral pars opercularis of the inferior frontal gyrus would predict better performance on the stop-signal task in a cohort of 110 children and adolescents 4–13 years of age, with one to four observations (totaling 232 observations). Cortical thickness of the bilateral opercular region was not related to inhibitory performance. However, independent of age, gender, and total cortical surface area, relatively larger cortical surface area of the bilateral opercular region of the inferior frontal gyrus was associated with better motor-inhibitory performance. Follow-up analyses showed a significant effect of surface area of the right pars opercularis, but no evidence for an effect of area of left pars opercularis, on motor-inhibitory performance. These findings are consistent with the previous work in adults showing that cortical morphology of the pars opercularis is related to inhibitory functioning. It also expands upon this literature by showing that, in contrast to earlier work highlighting the importance of cortical thickness of this region in adults, relative cortical surface area of the pars opercularis may be related to developing motor-inhibitory functions during childhood and adolescence. Relationships between cortical phenotypes and individual differences in behavioral measures may vary across the lifespan.

## Introduction

Research has long focused on the neural substrates of psychiatric disorders. However, in recent years, there has been a greater shift toward identifying neurobiological correlates of basic behavioral phenotypes that can be measured dimensionally and cut across disorders (Insel et al. 2010). One behavioral phenotype that has received attention is response inhibition (Casey et al. 1997; Liddle et al. 2001; Johnstone et al. 2007; Forstmann et al. 2008; Tamm et al. 2002; Newman et al. 2015a; Madsen et al. 2010). Motor response inhibition is typically defined as the ability to withhold a planned motor response to a stimulus or to stop an ongoing response (Aron et al. 2004). Impairment in this basic process has been most commonly associated with attention-deficit/hyperactivity disorder (ADHD) (Barkley 1997), though it has been associated with other psychiatric disorders as well, such as anxiety and mood disorders (Wright et al. 2014), and schizophrenia (Ethridge et al. 2014).

Response inhibition is most often measured using standardized, continuous-performance tasks such as the stop-signal paradigm (Logan and Cowan 1984) or a variant of the go/no-go task (GNG) (Conners et al. 2003; Rosvold et al. 1956). The ability to inhibit a preplanned motor response has been linked to a highly interconnected, predominantly right-lateralized circuit involving frontal, motor, and striatal regions (Chambers et al. 2009). According to one model, the inferior frontal gyrus (IFG) is thought to be the origin of a “stop” signal, inhibiting the motor response via direct stimulation of the subthalamic nucleus and resulting in inhibition of motor output of the thalamus (Chambers et al. 2009). This description of the neural system underlying response inhibition is supported by functional magnetic resonance imaging (fMRI) studies. Functional studies, both in adults and in clinical populations, implicate the IFG as a region involved in successful response inhibition (Aron and Poldrack 2006; Eagle et al. 2008). Some previous investigations into the functional correlates of cognitive control and response inhibition suggest gender differences in regional activation (Bell et al. 2006; Garavan et al. 2006; Liu et al. 2012; Weiss et al. 2003) and age by gender interactions during adolescence (Rubia et al. 2010, 2013).

Despite the extensive work on brain functional correlates of response inhibition in healthy populations, research on the relationship between response inhibition and cortical morphology is limited, particularly in developing children and adolescents. Several studies have addressed this indirectly by examining neuroanatomical differences between children with ADHD and comparison groups. The previous studies comparing children and adolescents with and without ADHD symptoms found thinner cortex in ADHD and a relationship between increased rate of cortical thinning and the severity of ADHD symptoms (Shaw et al. 2011, 2013; Batty et al. 2010; Proal et al. 2011). Shaw and colleagues argued their findings supported a dimensional approach to ADHD, where the disorder is considered one extreme of a continuum of a behavioral phenotype. In other words, rather than simply investigating binary groups of participants with or without a diagnosis, a better approach to studying typical development and the development of clinical disorders would be examining the entire range of cognitive and behavioral performance.

Madsen et al. (2010) used diffusion-weighted imaging to examine associations between stop-signal reaction time (SSRT) performance, which is operationally defined as the ability to withhold or cancel an initiated motor response, and white matter microstructure in children. They found that after controlling for age, better response inhibition was associated with higher fractional anisotropy in the white matter underlying the IFG. However, no studies have examined relationships of SSRT to both thickness and surface area of the IFG in this age group. In a study of young adults with or without a childhood diagnosis of ADHD, our group found that thinner cortex in the opercular region was related to better performance on a Go/No-go task, independent of ADHD status (Newman et al. 2015a, b). However, cortical surface area of the same region was unrelated to performance.

The relationships among surface area, thickness, and response inhibition observed in adults may not translate directly to brain–behavior relationships in children. Cortical surface area and cortical thickness show distinct developmental trajectories, which may be mediated by distinct developmental processes and distinct genetic influences (Panizzon et al. 2009; Brown et al. 2012; Jernigan et al. 2011; Chen et al. 2012). It is, therefore, also necessary to begin to investigate differences in these relationships as a function of age (Casey et al. 2014). Our group recently took this approach to determine neural architectural correlates of anxiety in typically developing children and adolescents (Newman et al. 2015b). We found that higher anxiety was associated with thinner cortex globally and decreased relative surface area of the ventromedial prefrontal cortex, but that the strength of these associations diminished with age. It is, therefore, reasonable to consider whether a similar age interaction may be present in any association between response inhibition and cortical morphology. In addition, our group found that relatively larger surface area of the anterior cingulate was positively related to better performance on a flanker task in children less than 12 years of age, but this relationship was not present in older adolescents examined in the same study (Fjell et al. 2012). Thus, it is reasonable to hypothesize that in our younger developing cohort, there may be a relationship between regional surface area and motor-inhibitory performance.

The current project aims to build on and extend our previous findings in adults (Newman et al. 2015a) by examining the relationship between both cortical thickness and cortical surface area and motor-inhibitory performance in typically developing children and adolescents. Due to the distinct developmental trajectories of cortical surface area and thickness (Brown et al. 2012; Wierenga et al. 2014), we may observe a different pattern of results relative to adults. Our primary hypotheses were (1) that apparent thinning of the pars opercularis of the inferior frontal gyrus would correspond to better performance, independent of age and gender, and (2) that a relatively larger surface area of the same region would correspond to better response inhibition, independent of age and gender. Given the inconsistent laterality of previous findings, we did not have strong hypotheses about laterality, and so for both primary hypotheses, we examined the bilateral pars opercularis. Contingent upon finding significant effects in the bilateral region of interest and in light of previous findings suggesting that these associations may differ as a function of age and/or gender, follow-up analyses examined age and gender interactions. Finally, we examined associations with the right and left pars opercularis separately.

## Methods

### Participants

Participants were part of the Pediatric Longitudinal Imaging, Neurocognition, and Genetics study at the University of California, San Diego. Prior to participation, participants under 7 years old provided verbal assent, participants over 7 years old provided written assent, and parents or guardians provided written consent after an oral description of the study was provided. Participants were required to understand directions presented in English and have normal or corrected-to-normal hearing and vision. Potential participants with neurological disorders, significantly preterm birth, a diagnosis of autism spectrum disorder, mental retardation, and/or head trauma with loss of consciousness lasting more than 30 min, or daily drug or alcohol use by the mother during pregnancy, were excluded.

**Fig. 1:**
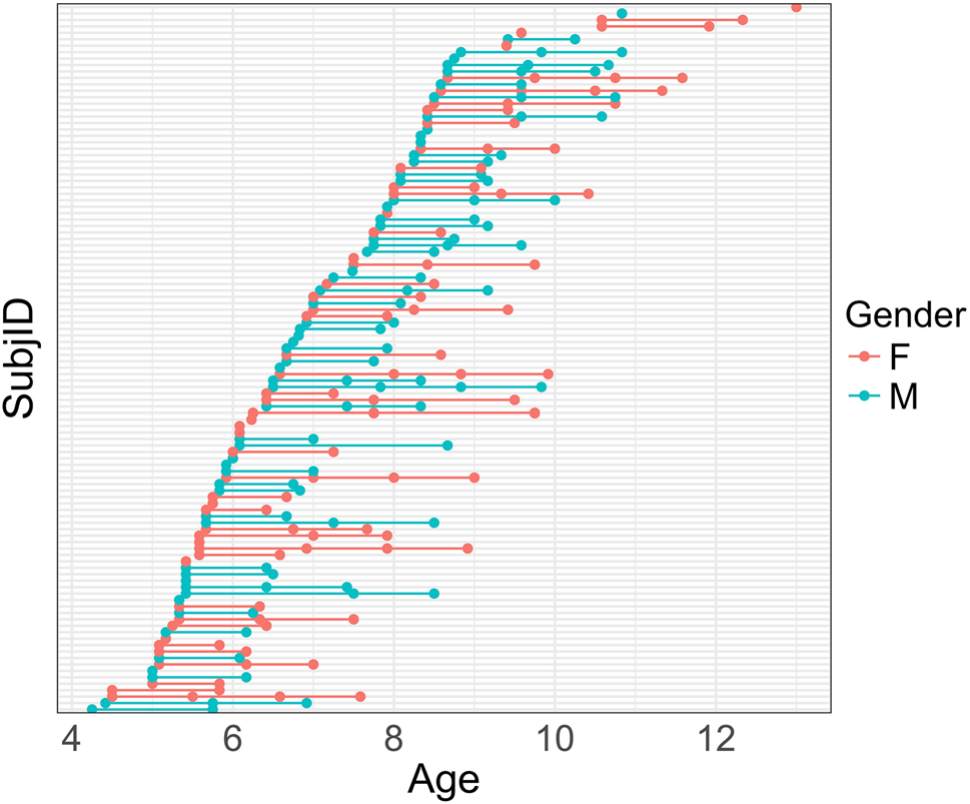
Summary of repeated-measures. Age is plotted on the *x*-axis, grouped by subject on the *y*-axis. Female participants are shown in *red*; male participants in *light blue*

The sample consisted of 110 typically developing children (59 male) between the ages of 4 and 13 years. Of these 110 participants, 82 had complete measurements for two visits, 32 had three visits, and 8 had four visits taken at approximately 1-year intervals, for a total of 232 visits. The average age of participants at the first visit was 6.9 years (SD 1.57 years, *n* = 110). At the second visit, the average age was 7.90 years (SD 1.45 years, *n* = 82), at the third visit 9.07 years (SD 1.31 years, *n* = 32), and the fourth visit 9.71 years (SD 1.30 years, *n* = 8) (see Table 1; Fig. 1).

**Table 1:** Summary of demographic and repeated-measures data

Demographics	Total	Male	Female
Number of participants	110	59	51
Age [mean (SD) in years]			
Baseline (*N* = 110)	6.9 (1.57)	6.92 (1.41)	6.87 (1.76)
Time point 2 (*N* = 82)	7.90 (1.45)	7.95 (1.29)	7.85 (1.63)
Time point 3 (*N* = 32)	9.07 (1.31)	9.26 (1.27)	8.90 (1.37)
Time point 4 (*N* = 8)	9.71 (1.30)	9.83 (0.00)	9.69 (1.41)
Stop-signal reaction time [mean (SD) in ms]			
Baseline (*N* = 110)	298.62 (114.33)	316.06 (129.68)	278.45 (90.64)
Time point 2 (*N* = 82)	258.50 (95.04)	267.00 (99.84)	248.65 (89.46)
Time point 3 (*N* = 32)	232.77 (98.38)	241.52 (121.76)	225.05 (75.13)
Time point 4 (*N* = 8)	232.47 (112.52)	238.80 (0.00)	231.56 (121.51)
Handedness (R/L/Amb/NA)^a^	83/14/9/4		
Race/ethnicity^b^			
Caucasian	54		
African American	5		
Hispanic/Latino	36		
Asian	13		
Pacific Islander	1		
American Indian	1		
Mixed race	22		
Other	3		

Number of participants, age, and stop-signal reaction time are outlined for the overall sample and also by male/female subgroups. Age and stop-signal reaction time means and standard deviations (SD) are given for each time point for the overall sample and by male/female subgroups. Handedness is reported for the overall sample (*R* right handed, *L* left handed, *Amb* ambidextrous, *NA* not reported). Race and ethnicity are reported for the overall sample

^a^Four participants did not identify handedness

^b^Participants were free to mark whichever race/ethnicity options they chose: if multiple races were checked, s/he was categorized as “Mixed race”; if none was selected, s/he was categorized as “Other”. Some participants marked only “Hispanic/Latino”, while others marked “Hispanic/Latino” in addition to a race. Therefore, the total number reported in each category does not sum to the total number of participants

### Stop-signal reaction time (SSRT)

**Fig. 2:**
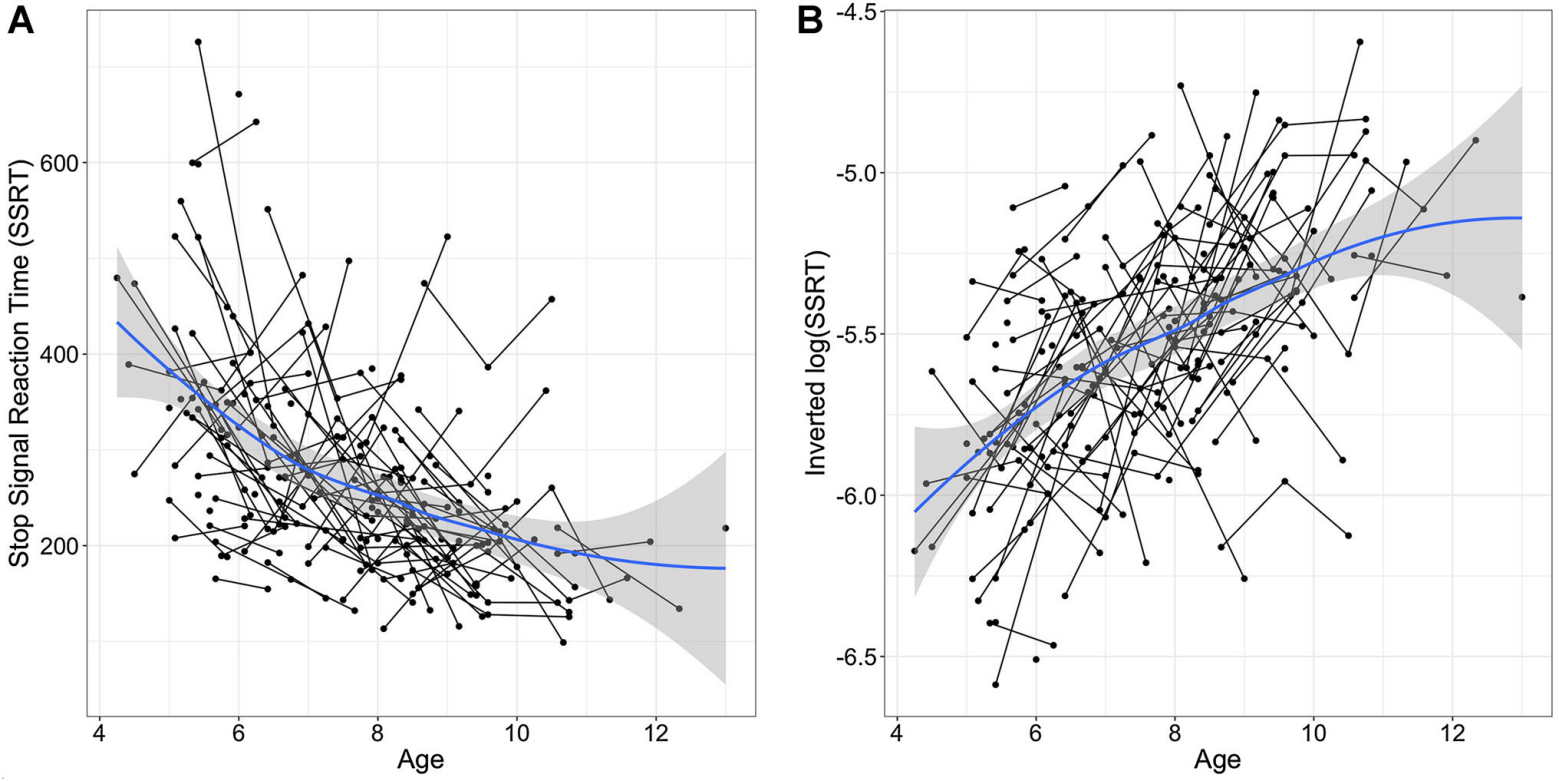
**a** Stop-signal reaction time (SSRT) as a function of age. Lower SSRT scores indicate better performance (i.e., faster reaction times). The *blue line* is smooth loess fit to the raw data, with *shaded* 95% confidence intervals around the mean at each point. **b** Inverted log(SSRT) scores as a function of age, where higher scores indicate better performance. Inverted log(SSRT) scores were used as the dependent measure in all models

We administered the stop-signal task from the Cambridge Neuropsychological Test Automated Battery (CANTAB, Cambridge Cognition Ltd., Cambridge, UK; Fray et al. 1996). While seated at a computer, participants rested the index finger of each hand on left and right response buttons. A fixation circle was presented for 500 ms, after which an arrow appeared in the center pointing either right or left. The participant was instructed to respond with the relevant response key (right or left) corresponding to the direction of the arrow, as quickly as possible. The stop-signal task is made up of ‘go’ trials (75%) and ‘stop’ trials (25%) presented over five blocks of 64 trials each. On the ‘stop’ trials, a tone is presented at a variable delay after the ‘go’ signal, indicating that the participant should withhold the response. A participant’s stop-signal delay (SSD) is the delay at which he/she can successfully withhold his/her response 50% of the time. The stop-signal reaction time (SSRT) is calculated for each participant by subtracting the SSD from the median reaction time on ‘go’ trials. This measure indicates the time each individual participant needs to refrain from executing a preplanned motor action upon presentation of a stop signal, with lower reaction times indicating better performance. For all behavioral and structural analyses, the logarithm of stop-signal reaction time was used as a variance stabilizing transformation. The log(SSRT) measure was then inverted, so that higher scores correspond to better performance allowing for more intuitive interpretation of results (see Fig. 2a, b).

### Neuroimaging

**Fig. 3:**
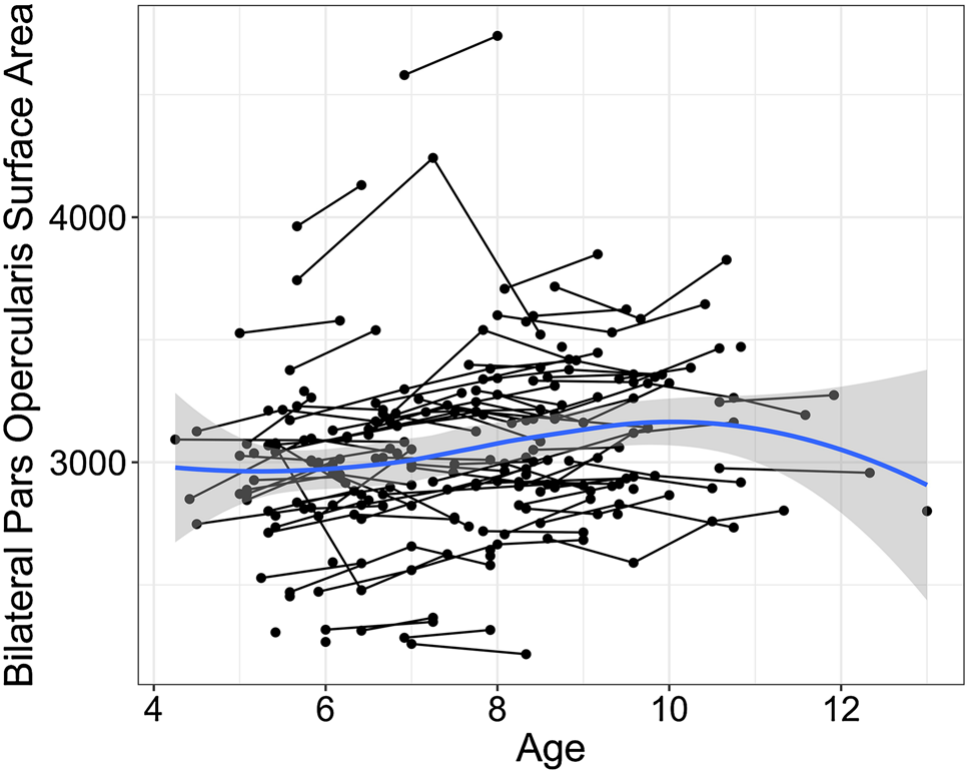
Bilateral pars opercularis surface area is shown on the *y*-axis and age is shown on the *x*-axis. The *blue line* is smooth loess fit to the raw data, with *shaded* 95% confidence intervals for the mean at each point

All neuroimaging data were collected at UC San Diego using the PING protocol (see Jernigan et al. 2016b for details). This is a multiple modality, high-resolution magnetic resonance imaging (MRI) protocol during which participants underwent a 1-h imaging session including acquisition of TI, T2, and diffusion-weighted images. All data were evaluated for quality at multiple stages during processing, including registration, motion correction, and removal of artifacts. Automated protocols available in Freesurfer (Fischl 2004) in addition to analyses developed at UC San Diego Multimodal Imaging Laboratory were used for processing and morphometric analysis. The right and left pars opercularis were extracted using the Desikan atlas available in Freesurfer (Desikan et al. 2006). To create the bilateral pars opercularis thickness region of interest, the right and left pars opercularis measures were averaged. To create the bilateral pars opercularis surface area region of interest, the right and left pars opercularis areas were added together (see Fig. 3). Post hoc cortical surface-based mapping analyses relied upon nonlinear, surface-based registration constrained by cortical folding patterns (Fischl et al. 1999), and used surface-constrained, iterative smoothing with 705 iterations, equivalent to ~33 mm full width at half maximum (Hagler et al. 2006).

### Analysis

We tested the primary hypotheses with two separate region-of-interest analyses using longitudinal mixed-effects regression models to predict inverted log(SSRT) scores from cortical thickness and surface area of the pars opercularis. Analyses were carried out using the nlme package in R with a covariance structure of AR(1). Bilateral pars opercularis thickness and surface area were both centered (demeaned) prior to analysis and entered as fixed effects. Preliminary analyses investigating age and gender effects on stop-signal performance indicated an effect of gender, which was then included in all primary models investigating region-of-interest effects. Age and gender were centered (with female coded as negative and male as positive) and entered as fixed effects in the model, while subject ID was entered as a random effect. Total cortical surface area was centered and also included as a covariate in the surface area model to estimate the effect of relative surface area of the pars opercularis. Scanner was included as a covariate of no interest. For each of the two main hypotheses, a Bonferroni-corrected *p* value of 0.025 was used as the threshold for significance.

If either of the primary models examining bilateral pars opercularis was significant, interactions between age, gender, and the bilateral pars opercularis were included in a later model to determine if significant interactions were present. Finally, the left and right pars opercularis were examined in separate models.

## Results

The average stop-signal reaction time (SSRT) for all participants at baseline was 298.62 ms (SD 114.33 ms, *n* = 110). At the second visit, the average score was 258.50 ms (SD 95.04 ms, *n* = 82), at the third visit, the average score was 232.77 ms (SD 98.38 ms, *n* = 32), and at the fourth visit, the average score was 232.47 ms (SD 112.52 ms, *n* = 8) (see Table 1).

The hypothesis that cortical thickness in the bilateral pars opercularis would significantly predict inverted log(SSRT) scores was tested using a linear mixed-effects model covarying for age and gender (Table 2). Age and gender were both significant predictors of inverted log(SSRT) scores, with older participants (*t* = 7.62, *p* < 0.001) and females (*t* = −2.09, *p* = 0.04) performing better. However, there was no significant relationship between bilateral pars opercularis thickness and inverted log(SSRT) scores (*t* = −1.33, *p* = 0.19).

**Table 2:** Inverted log(SSRT) scores were predicted using a linear mixed-effects model

Fixed effects	*B* value	*t* value	*p* value
Age^a^	0.10	7.62	0.0000***
Gender^a^	−0.11	−2.09	0.0389*
Bilateral pars opercularis thickness^a^	−0.27	−1.33	0.1853
Scanner	0.12	1.37	0.1682

Predictors included bilateral pars opercularis thickness and covariates were age, gender, and scanner. Where noted, predictors were centered (demeaned)

Random effect: subject

^a^Predictor has been centered (demeaned), *p<.05, ***p<.001

The hypothesis that relative surface area in the bilateral pars opercularis would significantly predict inverted log(SSRT) scores was tested with a similar linear mixed-effects model with total surface area as an additional covariate (Table 3). We found a significant, positive relationship between bilateral pars opercularis surface area and inverted log(SSRT) scores (*t* = 2.53, *p* = 0.01), where larger surface area was associated with better performance on the stop-signal reaction time task. Consistent with the thickness model, older participants (*t* = 7.76, *p* < 0.001) performed better. Total cortical surface area was not related to inverted log(SSRT) scores.

**Table 3:** Inverted log(SSRT) scores were predicted using a linear mixed-effects model

Fixed effects	*B* value	*t* value	*p* value
Age^a^	0.10	7.76	0.0000***
Gender^a^	−0.11	−1.94	0.0553
Bilateral pars opercularis surface area^a^	0.00	2.53	0.0127*
Total cortical surface area^a^	−0.00	−0.84	0.4049
Scanner	0.10	1.15	0.2535

Predictors included bilateral pars opercularis surface area, and covariates were age, gender, total cortical surface area, and scanner. Where noted, predictors were centered (demeaned)

Random effect: subject

^a^Predictor has been centered (demeaned), *p<.05, ***p<.001

Because the model examining bilateral pars opercularis surface area was significant, an additional follow-up analysis examined interactions between age, gender, and bilateral pars opercularis surface area (Table 4). In a model including all interactions between age, gender, and bilateral pars opercularis surface area, there were no significant interaction terms. We then performed follow-up analyses investigating left and right pars opercularis surface area separately (Table 5). Right pars opercularis surface area was positively related to better SSRT performance (*t* = 2.60, *p* = 0.01), but the effect of left pars opercularis surface area was not significant (*t* = 1.21, *p* = 0.23). Of note, we had two main a priori hypotheses regarding both bilateral pars opercularis surface area and thickness, and thus, we corrected for two statistical tests (see “Analysis”, above). The additional models exploring interactions between age, gender, and bilateral pars opercularis surface area and then left and right pars opercularis were not corrected for multiple comparisons as they were post hoc tests contingent upon prior significant effects.

**Table 4:** Inverted log(SSRT) scores were predicted using a linear mixed-effects model

Fixed effects	*B* value	*t* value	*p* value
Age^a^	0.10	7.20	0.0000***
Gender^a^	−0.11	−1.97	0.0516
Bilateral pars opercularis surface area^a^	0.00	2.27	0.0250*
Total cortical surface area^a^	−0.00	−0.91	0.3664
Age^a^ × gender^a^	0.04	1.53	0.1289
Age^a^ × bilateral pars operc^a^	−0.00	−0.56	0.5780
Gender^a^ × bilateral pars operc^a^	0.00	0.81	0.4191
Age^a^ × gender^a^ × bilateral pars operc^a^	0.00	1.49	0.1384
Scanner	0.10	1.14	0.2574

Predictors included bilateral pars opercularis surface area and covariates were age, gender, total cortical surface area, and scanner. All interaction terms for age, gender, and bilateral pars opercularis surface area were included. Where noted, predictors were centered (demeaned)

Random effect: subject

^a^Predictor has been centered (demeaned), *p<.05, ***p<.001

**Table 5:** Inverted log(SSRT) scores were predicted using linear mixed-effects models

	*B* value	*t* value	*p* value
(a) Left pars opercularis model			
Age^a^	0.11	7.92	0.0000***
Gender^a^	−0.12	−1.98	0.0505
Left pars opercularis surface area^a^	0.00	1.21	0.2295
Total cortical surface area^a^	0.00	0.10	0.9196
Scanner	0.11	1.18	0.2399
(b) Right pars opercularis model			
Age^a^	0.10	7.75	0.0000***
Gender^a^	−0.10	−1.62	0.1084
Right pars opercularis surface area^a^	0.00	2.60	0.0106*
Total cortical surface area^a^	−0.00	−0.73	0.4659
Scanner	0.11	1.23	0.2214

(a) Surface area of the left pars opercularis with covariates age, gender, total cortical surface area, and scanner. (b) Surface area of the right pars opercularis with covariates age, gender, total cortical surface area, and scanner. Where noted, predictors were centered (demeaned)

Random effect: subject

^a^Predictor has been centered (demeaned), *p<.05, ***p<.001

**Fig. 4:**
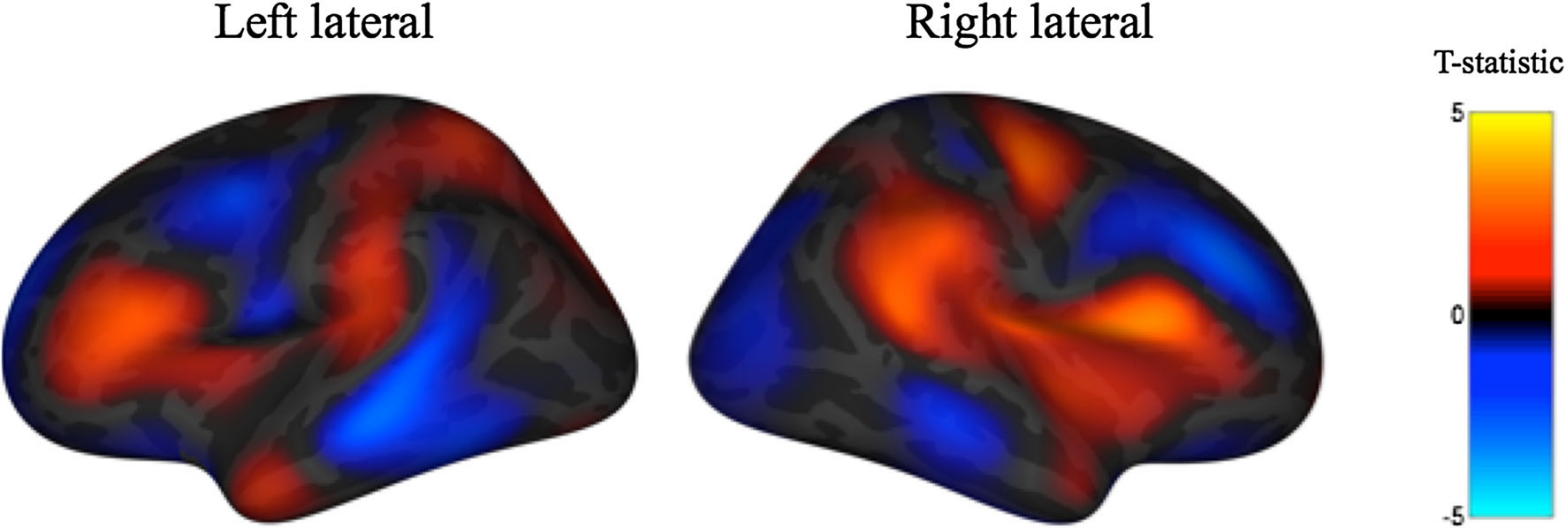
Post hoc exploratory vertex-wise maps depicting effect of inverted log(SSRT) on cortical surface area. Covariates include demeaned age and gender, and scanner. Heat maps reflect the *t*-statistic values on a scale from −5 to 5

To visualize the relationship between age and surface area, we created post hoc, vertex-wise maps of (uncorrected) *t*-statistics for the surface area effects on inverted log(SSRT) scores, controlling for age, gender, total cortical surface area (which were all demeaned, as above), and scanner. For this visualization, we used the baseline observations only (*N* = 110). The color scale codes *t*-statistic values, ranging from −5 to 5 with the boundary between warm and cool colors at zero. As reported above, there appears to be an association between relative surface area of the pars opercularis and SSRT performance. In addition, the visualization suggests that there may be modest positive and negative associations across both the left and right cortical surfaces in other regions (see Fig. 4).

## Discussion

This study investigated the relationship between variation in regional cortical morphology and performance variability on a response-inhibition task in a longitudinal, typically developing cohort. Based on the previous work, we focused our analyses on the pars opercularis of the inferior frontal gyrus (Aron and Poldrack 2006; Chambers et al. 2009; Eagle et al. 2008; Newman et al. 2015a). The primary finding from this study was that greater relative cortical surface area in the bilateral pars opercularis was associated with better response inhibition. Thickness in this region showed no significant relationship with inhibitory functioning.

These results stand in contrast to a recent study in an adult sample showing that cortical thickness in this region, but not surface area, was associated with response inhibition. Specifically, better inhibitory functioning was associated with thinner cortex in the IFG. A number of methodological differences could contribute to the differences observed in these two studies. First, the previous study used percentage of commission errors on a go/no-go task to measure response inhibition, whereas the current study used an estimate of time needed to successfully inhibit a response on a stop-signal task. Second, the previous sample was comprised entirely of young adults, whereas the current sample was comprised of children ranging in age from 4 to 13. Third, the sample in the previous study was comprised of individuals diagnosed with ADHD as well as comparison participants, and, therefore, reflected wide variability in inhibitory functioning. The current sample was of typically developing children.

While there are differences in task demands between the go/no-go and stop-signal tasks, functional studies have linked both to the function of the IFG (Chikazoe et al. 2007; Chikazoe 2010; Aron et al. 2015). It may be that age plays a larger role in the different relationships observed between the pars opercularis and performance observed in these two studies. In a recent cross-sectional study linking anxiety to regional cortical morphology in children and adolescents, our group found that regional surface area, but not regional thickness, predicted behavior (Newman et al. 2015b). Specifically, greater relative surface area expansion of the ventromedial prefrontal cortex was associated with lower self-reported anxiety.

Recent studies have shown that there is very little overlap between the genetic factors that influence surface area and thickness, although they are both highly heritable (Chen et al. 2011, 2012; Panizzon et al. 2009), and their developmental trajectories are markedly different (Brown et al. 2012; Jernigan et al. 2016a). On average, surface area increases steadily until middle childhood and begins to taper off in adolescence and early adulthood, and these changes occur at different rates in different regions of the cortex. In contrast, cortical thickness decreases consistently and continuously over the course of development (Brown et al. 2012; Walhovd et al. 2016). Therefore, future work should aim to assess the differential contributions of regional surface area and thickness to cognitive performance in developing cohorts.

The exploratory vertex-wise surface maps of the relationship between regional surface area and response inhibition show the predicted bilateral effect in the pars opercularis. These maps provide additional information to readers about the degree of variability across the cortical surface in the direction and magnitude of the relationship between relative surface area expansion and SSRT performance.

## Conclusions and limitations

In this study, we examined a large number of typically developing children in a longitudinal cohort to determine whether we could confirm an association between regional morphology of the inferior frontal gyrus and performance on the stop-signal task. The results suggested a relationship between regional cortical surface area of the pars opercularis and performance on this motor-inhibitory task. In contrast to our group’s earlier work highlighting the relationship between the cortical thickness of this region and inhibitory control task performance in adults (Newman et al. 2015a), it appears that the relative cortical surface area of the pars opercularis may be especially important for the development of inhibitory control, although directly assessing this relationship requires further examination. This highlights the possibility that different cortical phenotypes may show differential or unique relationships to behavioral functions at different points during development. However, among the many possible influences on developing brain structure and response inhibition, this study evaluated only age and gender as covariates. In addition to age and gender, there is evidence that genetics, experience, socioeconomic status, and many other factors could affect both measures of brain structure and cognitive performance (Chen et al. 2012; Noble et al. 2015). Future analyses should also evaluate other covariates thought to relate to both structural brain development and response inhibition to form a more complete picture of the factors influencing these relationships.

## Abbreviations

ADHD: Attention deficit hyperactivity disorder
GNG: Go/no-go
IFG: Inferior frontal gyrus
fMRI: Function magnetic resonance imaging
SSRT: Stop-signal reaction time
SSD: Stop-signal delay
MRI: Magnetic resonance imaging
